# Use of microaggregate blood filters instead of leukocyte reduction filters to purify salvaged, autologous blood for re-transfusion during obstetric surgery

**DOI:** 10.1007/s00540-013-1579-7

**Published:** 2013-03-26

**Authors:** Ju Mizuno

**Affiliations:** Department of Anesthesiology and the Intensive Care Unit, Teikyo University School of Medicine, 2-11-1 Kaga, Itabashi-ku, Tokyo, 173-8605 Japan

To the Editor:

Intraoperative, salvaged, autologous blood re-transfusions are especially useful to treat profuse hemorrhage that occurs during obstetric surgeries [1]. A prominent concern with such autotransfusions is the risk that the relatively large pore size (170–260 μm) of standard blood transfusion filters may not eliminate all amniotic and fetal materials and other contaminants from the salvaged, autologous blood where they can cause amniotic fluid embolization (AFE).

Use of leukocyte reduction filters (LRFs) with 10–50 μm pore size during salvage of autologous blood in cesarean sections appears to be able to eliminate amniotic and fetal particulate substances such as fetal squamous cells [2], and thereby prevent AFE. Intraoperative autotransfusions with LRFs, therefore, represent a promising technique for the treatment of profuse hemorrhage in obstetric surgeries. However, since 2007, the Japanese Red Cross Blood Center has provided pre-storage leukocyte-reduced red cell concentrate containing a citrate–phosphate–dextrose solution instead of an acid–citrate–dextrose solution as anticoagulant in which the leukocytes were reduced soon after collection. LRFs have not been made since 2006, and have not been supplied since 2009 by Japanese manufacturers.

Microaggregate blood filters (MBFs) with 10–40 μm pore size are able to eliminate larger-diameter, retained, blood component microaggregate materials and non-blood, particulate matter that is potentially harmful to the recipients. In other words, MBFs assure the patient protection from microaggregate materials, clots, and particulate debris that can cause pulmonary embolization [3].

We managed a case with a re-transfusion of salvaged, autologous blood using a Pall SQ40^®^ (Pall, Tokyo, Japan) MBF with 40 μm pore size instead of LRF for massive hemorrhage during obstetric surgery [4]. Finally, abortion and total abdominal hysterectomy were performed and no AFE or other complications occurred.

The Pall SQ40^®^ MBF is a free-flow, low priming volume device which provides a 40 μm rated filter and media pleating for the elimination of potentially harmful blood component microaggregates and non-blood component particulate matter [5]. Pall’s 40 μm rated polyester screen media is process-controlled and monitored to ensure uniformity of pore size to assure maximum patient protection from microaggregates, clots, and particulate debris.

Further, screen-type MBFs are able to maintain a relatively high speed of transfusion, because of their larger total filter area. The total filter area of Pall’s screen-type MBF (160 cm^2^) provides up to twelve times the typical first surface area of a depth-type MBF. This feature reduces the number of administration set changes required for rapid transfusion, particularly for blood components with a large debris load such as salvaged blood components. In massive obstetric hemorrhage, rapid re-transfusions are required for resuscitation. Since LRFs limit the speed of transfusion, it is sometimes necessary to suspend or forego the use of an LRF, and to use screen-type MBFs, instead, in order to increase the speed of transfusion.

In conclusion, MBFs instead of LRFs should be available and ready to use to purify salvaged, autologous blood for re-transfusion to treat profuse hemorrhage during obstetric surgeries, and to prevent AFE.

## References

[1] Allam J, Cox M, Yentis SM (2008). Cell salvage in obstetrics. Int J Obstet Anesth.

[2] Waters JH, Biscotti C, Potter PS, Phillipson E (2000). Amniotic fluid removal during cell salvage in the cesarean section patient. Anesthesiology.

[3] Reul GJ, Beall AC, Greenberg SD (1974). Protection of the pulmonary microvasculature by fine screen blood filtration. Chest.

[4] Morita Y, Mizuno J, Takada S, Yunokawa S, Morita S (2009). Massive hemorrhage during abdominal total hysterectomy in a patient with placenta percreta. Masui.

[5] Cullen DJ, Kunsman J, Caldera D, Dennis RC, Valeri CR (1980). Comparative evaluation of new fine-screen filters: effects on blood flow rate and microaggregate removal. Anesthesiology.

